# Mechanisms of Immunity in Post-Exposure Vaccination against Ebola Virus Infection

**DOI:** 10.1371/journal.pone.0118434

**Published:** 2015-03-18

**Authors:** Steven B. Bradfute, Scott M. Anthony, Kelly S. Stuthman, Natarajan Ayithan, Prafullakumar Tailor, Carl I. Shaia, Mike Bray, Keiko Ozato, Sina Bavari

**Affiliations:** 1 United States Army Medical Research Institute of Infectious Diseases, Fort Detrick, Maryland, United States of America; 2 Laboratory of Molecular Growth Regulation, National Institute of Child Health and Human Development, National Institutes of Health, Bethesda, Maryland, United States of America; 3 National Institute of Immunology, New Delhi, India; 4 Division of Clinical Research, National Institute of Allergy and Infectious Diseases, National Institutes of Health, Bethesda, Maryland, United States of America; George Mason University, UNITED STATES

## Abstract

Ebolaviruses can cause severe hemorrhagic fever that is characterized by rapid viral replication, coagulopathy, inflammation, and high lethality rates. Although there is no clinically proven vaccine or treatment for Ebola virus infection, a virus-like particle (VLP) vaccine is effective in mice, guinea pigs, and non-human primates when given pre-infection. In this work, we report that VLPs protect Ebola virus-infected mice when given 24 hours post-infection. Analysis of cytokine expression in serum revealed a decrease in pro-inflammatory cytokine and chemokine levels in mice given VLPs post-exposure compared to infected, untreated mice. Using knockout mice, we show that VLP-mediated post-exposure protection requires perforin, B cells, macrophages, conventional dendritic cells (cDCs), and either CD4+ or CD8+ T cells. Protection was Ebola virus-specific, as marburgvirus VLPs did not protect Ebola virus-infected mice. Increased antibody production in VLP-treated mice correlated with protection, and macrophages were required for this increased production. However, NK cells, IFN-gamma, and TNF-alpha were not required for post-exposure-mediated protection. These data suggest that a non-replicating Ebola virus vaccine can provide post-exposure protection and that the mechanisms of immune protection in this setting require both increased antibody production and generation of cytotoxic T cells.

## Introduction

Although mechanisms of immunity in pre-exposure vaccination against pathogens are often studied, less information is available on how post-exposure vaccination protects from infection. For many pathogens, there are no post-exposure vaccination therapies available. Ebolaviruses, members of the *filoviridae*, can cause hemorrhagic fever resulting in death in 20–88% of infected humans [1–2]. Zaire ebolavirus (EBOV) is the most prevalent ebolavirus species in humans [1]. The disease is characterized by high fever, rapid viral propagation, hemorrhage, and dysregulated cytokine production [1]. A number of filovirus vaccine platforms have been shown to be efficacious in rodent and non-human primate models when given as a pre-exposure vaccine, including those derived from adenovirus (AdV), vesicular stomatitis virus (VSV), parainfluenza virus, plasmid DNA, and Venezuelan Equine Encephalitis Virus replicon (VRP)-based platforms [3–7]. Additionally, virus-like particles (VLPs) have been shown to protect rodents and non-human primates from filovirus infection [8–10].

However, only a few studies have analyzed mechanisms of immunity in post-exposure protection mediated by filovirus vaccines. An AdV-based EBOV vaccine protects mice when given 30 minutes after infection [11], and post-exposure VSV vaccination protects mice and partially protects guinea pigs when given 24 hours after EBOV infection [12]. Most notably, the VSV platform has shown partial protection against ebolaviruses and complete protection against marburgviruses (another member of the *filoviridae*) when given to non-human primates 20–30 minutes after challenge; additional studies showed partial protection in non-human primates 24 or 48 hours after marburgvirus infection [12–15]. Antibody production is increased in surviving filovirus-infected non-human primates following VSV post-exposure treatment [12–14], but it is unknown if this is responsible for protection in this setting. T cell responses in these studies were either undetectable or not measured. It has been shown that transfer of specific polyclonal or monoclonal antibody preparations protects non-human primates after EBOV infection [16–20], strongly suggesting that induction of antibody responses may be required for successful post-exposure vaccination. Other experimental post-exposure treatments for EBOV include antisense therapies meant to inhibit viral replication [21–22]. The in-depth mechanisms of immunity in post-exposure vaccination is therefore not well-described, and there have been no mechanistic reports of post-exposure protection in a non-replicating EBOV vaccine.

We took advantage of the mouse model of EBOV infection to demonstrate the efficacy and immune mechanisms of protection in post-exposure, VLP-based vaccination. In our accompanying paper [69], we show that VLPs caused early induction of type I interferon pathways in infected mice, and resulted in decreased systemic inflammatory cytokine production. Here we report that protection was dependent on B cells and cytotoxic T cells, and correlated with increased antibody production. Together these data suggest that a non-replicating VLP vaccine given post-EBOV exposure protects by inducing early type I IFN responses, which leads to decreased systemic inflammation and enhanced adaptive immune responses.

## Materials and Methods

### Ethics statement

Research was conducted in compliance with the Animal Welfare Act and other federal statutes and regulations relating to animals and experiments involving animals and adheres to principles stated in the guide for the Care and Use of Laboratory Animals, National Research Council, 1996. The facility where this research was conducted is fully accredited by the Association for Assessment and Accreditation of Laboratory Animal Care International. The IACUC committee approving this protocol is the United States Army Medical Research Institute of Infectious Diseases (USAMRIID) IACUC. Animals were monitored at least once daily, and their status was evaluated according to an Intervention Scoresheet approved by USAMRIID IACUC. Monitoring increased to three times daily if the animals were given a score of three or four. Euthanization was by CO_2_ inhalation followed by confirmatory cervical dislocation. Analgesics and anesthetics were not used in this study and animals were euthanized for humane purposes if they reached a score of five or more, which would be indicated if the animals exhibited ruffled fur, weakness, unresponsiveness, and/or difficulty walking. Otherwise, animals were euthanized at the end of the study.

### Mice and infections

All EBOV-infected cells [9] and mice were handled under maximum containment in a biosafety level (BSL)-4 laboratory at the U.S. Army Medical Research Institute of Infectious Diseases (Frederick, MD, USA). Six-10 week old C57Bl/6 mice were used in experiments and were purchased from the National Cancer Institute (Frederick, MD, USA). Mice lacking perforin, CD4, CD8a, TCRa, IFN-gamma, TNF-alpha, gamma/delta T cell receptor, or B cells (mice lacking IgM heavy chain), as well as CD11c-DTR, CD11b-DTR, and SCID mice, were purchased from The Jackson Laboratory (Bar Harbor, Maine, USA). Mice were infected intraperitoneally (i.p.) with ~1000 pfu (~3,000 LD50) of mouse-adapted EBOV [23]. This dose was chosen since 1000 pfu is the standard dose of lethal EBOV infection in animal models; our analysis of this particular batch of mouse-adapted EBOV showed that 1000 pfu was ~3,000 LD50.

### Virus-Like Particles

VLPs were composed of EBOV (or Marburg) GP, NP, and VP40, and were generated in mammalian 293T cells as reported elsewhere [24]. Mice received 50 ug of VLP diluted in PBS i.p. 24 hours after infection (day +1) unless otherwise noted. In some experiments, VLPs composed of VP40 alone or VP40 and NP VLPs were generated and administered in a similar manner as above.

### Depletion of immune components

Antibody-mediated depletions were performed by injecting functional grade preparations i.p. into mice. All antibodies were purchased from eBioscience (San Diego, CA, USA) unless otherwise indicated. For depletion of lymphocytes, mice were injected i.p. with 300 ug of antibody of clone PK136 (anti-NK1.1) [25], clone GK1.5 (anti-CD4) [26], clone 53–6.7 (anti-CD8a) [27], or clone 30-H12 (anti-CD90.2) [28], or the appropriate isotype controls (rat IgG2a, rat IgG2b, or mouse IgG2a)) on days -3, -1, +3, and + 8. For depletion of TNF-alpha, mice were injected i.p. on Day 0 with 1 mg of anti-TNF alpha antibody (clone MP6-XT22) [29]. For depletion of IFN-gamma, mice were injected i.p. with 300 ug anti-IFN-gamma antibody (clone XMG1.2) or rat IgG1 isotype control on days-1, +1, +3, and +6 [30]. For depletion of conventional DCs (cDC) [31], CD11c-DTR or control wild-type mice were injected i.p. with 100 ng diphtheria toxin approximately 4 hours after Ebola infection. For depletion of CD11b+ cells [32–33], CD11b-DTR or control wild-type mice were injected with 500 ng of diphtheria toxin 48 hours and 4 hours prior to EBOV infection. Phagocytes were depleted by i.p. injection of 200 ul of clodronate-encapsulated liposomes (Encapsula) 24 hours prior to infection [34]; control mice received PBS-encapsulated liposomes.

### Cytokine assays and viral titers

Cytokine levels were measured in the mouse serum by using cytometric bead array flex sets (BD Biosciences) according to the manufacturer’s instructions. Viral titers were determined by standard plaque assay on Vero E6 cells as previously described [23].

### Histology

Tissues were harvested, fixed in 10% formalin for 28 days, and processed according to standard methods [23]. Paraffin sections were stained for hematoxylin and eosin (H&E), terminal deoxynucleotidyl transferase dUTP nick end labeling (TUNEL) (Chemicon, Temecula, CA, USA), or EBOV antigen.

### DC preparations

Spleens were injected with Liberase CI (Roche, Indianapolis, IN, USA) and incubated at 37C for 30 minutes, after which they were meshed through a 70-micron strainer and washed. The pellets were resuspended in 30% BSA in PBS, overlayed with PBS/0.1% BSA, and spun for 15 minutes at 9,500g at 4C. The low-density cells at the interface were collected, washed, and used for further analysis.

### ELISA

ELISAs were performed as described [24]. Briefly, plates were coated with irradiated EBOV, and blocked with 5% milk for two hours. Plates were washed and sera was added in serial 2-fold dilutions, in duplicate, and incubated for two hours. After four washes with PBS/0.1% Tween20, peroxidase-conjugated anti-mouse IgG or IgM antibody was added for 1 hour, after which plates were washed four times with PBS/0.1% Tween20, and peroxidase activity was detected using Sure Blue detection reagent and stop solution (KPL, Gaithersburg, MD, USA). Positive samples were defined as having greater than 2 times the O.D. (at 450 nm) of sera from uninfected day 0 mice.

### Statistics

Statistical analysis in the viremia, cytokine, and antibody studies were performed using a two-tailed student’s t-test.

## Results

### VLPs protect mice when given 24 hours after EBOV infection

**Fig 1:**
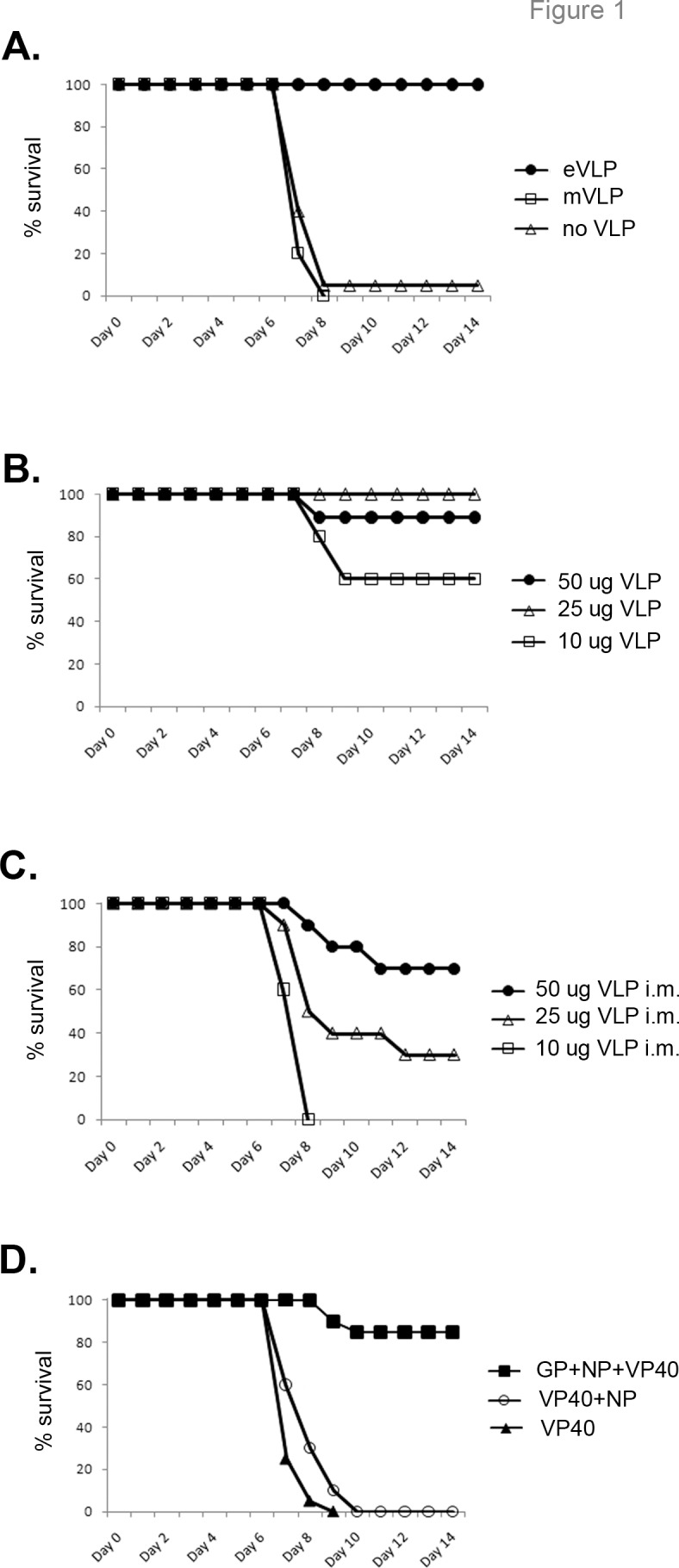
Post-exposure VLP treatment protects mice from EBOV infection. **(A)** Mice were infected i.p. with ~1,000 pfu mouse-adapted EBOV virus and 24 hours later were treated i.p. with 50 ug EBOV VLPs (GP+NP+VP40), Marburg VLP, or left untreated. EBOV-VLP-treated mice survived, whereas Marburg VLP-treated mice did not survive, and did not have delayed time-to-death compared to untreated mice. n = 10 for eVLP and mVLP; n = 20 for no VLP. (**B)** Mice were infected with EBOV and treated i.p. with different doses of EBOV VLPs 24 hours post-infection. n = 10. (**C)** Mice were infected with EBOV and treated intramuscularly with different doses of VLPs 24 hours post-infection. Less protection was seen with i.m. injection than i.p. treatment at lower doses. n = 10. (**D)** VLPs made from VP40 alone or VP40+NP did not protect mice when given i.p. 24 hours post-exposure. VLPs made from VP40+GP had some protection, but less than the trivalent VP40+GP+NP. n = 20 for VP40; n = 29 for VP40+NP; n = 20 for GP+NP+VP40.

Previous work has shown that VLPs are protective against EBOV when given either as a typical pre-exposure vaccine regimen [9] or 1–3 days prior to infection [35] in mice. However, no work has been published testing VLPs as a post-infection therapeutic. To address this, mice were infected with mouse-adapted EBOV (EBOV) and treated with VLPs composed of VP40, NP, and GP_1,2_ 24 hours post-infection. As shown in Fig. 1A, 50 ug of EBOV VLPs protected mice when given 24 hours after EBOV infection. Survival ranged from 70–100%, with an average of 93% (n = 103), compared to 9% survival with no VLP treatment (n = 146). This protection was antigen-specific, as mice treated with VLPs derived from marburgvirus (another filovirus) succumbed to EBOV infection (Fig. 1A). This data strongly suggested that protection was EBOV-specific, and not simply due to non-specific induction of immune responses. Post-exposure protection also occurred with lower doses of VLPs (Fig. 1B). VLPs were protective whether administered via the intraperitoneal or the intramuscular route, although protection was achieved at lower doses via the intraperitoneal route (Fig. 1C). A dose of 50 ug given intraperitoneally was chosen as a standard dose instead of using lower doses to err on the side of caution. VLP containing VP40 and NP, or VLP containing VP40 alone, did not offer any protection when given post-exposure (Fig. 1D), suggesting that the inclusion of GP was vital for protection. Therefore, the trivalent (VP40, NP, GP_1,2_) VLP administered intraperitoneally was most effective in generating protection post-exposure, and was subsequently used in the following studies.

### Post-exposure VLP-treated mice have reduced histological pathogenesis

**Fig 2:**
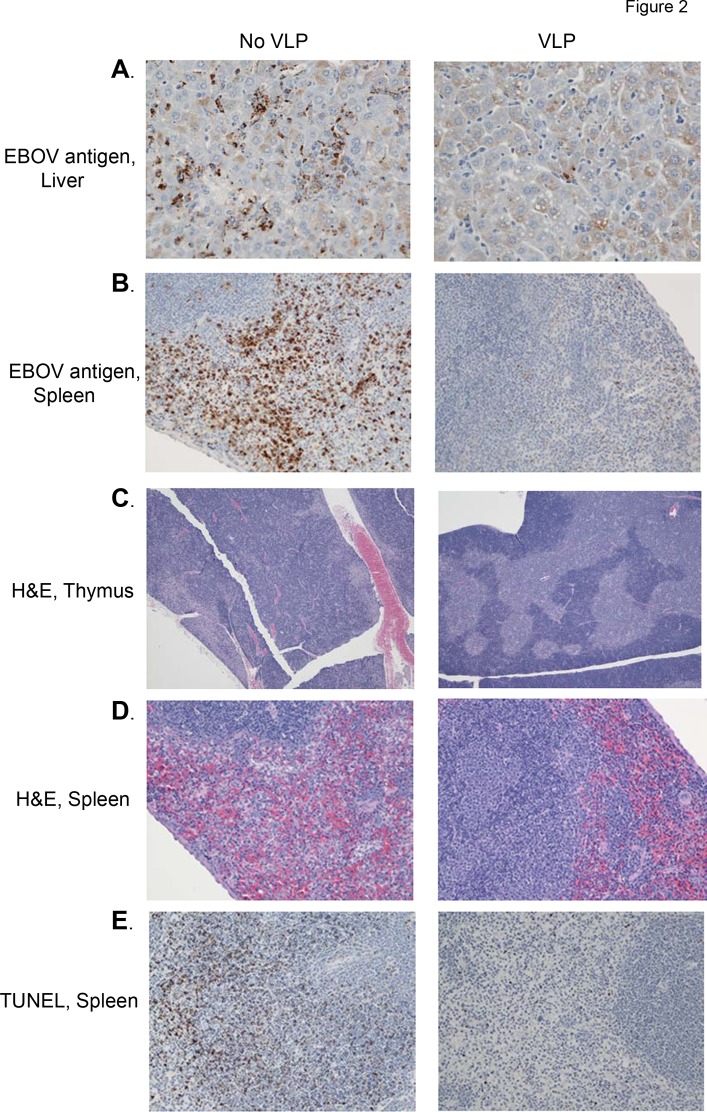
Decreased pathology of VLP-treated mice. Mice were infected with EBOV and left untreated (no VLPs) or were treated with 50 ug VLPs i.p. 24 hours after infection. On day 7 post-infection, tissues were harvested, fixed in formalin, processed, and analyzed. Three mice were harvested at each time point, and pictures show representative samples. **(A)** Staining for EBOV antigen (brown) in liver. Note reduction in virus in VLP-treated mice. Magnification 400x. **(B)** Staining for EBOV antigen in spleen. Similar findings as in liver. Magnification 200x. **(C)** H&E staining of thymus. Note the lack of architecture in the untreated thymus. Magnification 40x. **(D)** H&E staining in spleen, with lack of architecture in untreated spleen. Magnification 200x. **(E)** TUNEL staining in spleen. Untreated mice show increased apoptosis compared to VLP-treated mice. Magnification 200x.

Tissues from Day 0 (uninfected), Day 3, Day 5, and Day 7 EBOV-infected mice, either treated with VLPs 24 hours after infection or untreated, were harvested and analyzed for EBOV antigen distribution, apoptosis (via TUNEL staining), and H&E staining. The histopathologic findings in the liver, spleen, thymus, and lymph nodes of infected, untreated mice were similar to what has been described previously [23, 36]. However, mice receiving VLPs demonstrated less histologic evidence of disease on Days 3, 5 and 7 post-challenge than untreated mice (Fig. 2 and S1 and S2 Figs.). Day 7 VLP-treated samples clearly exhibited decreased viral antigen in the spleen and liver compared to untreated mice (Fig. 2A-B). Additionally, H&E staining of Day 7 thymus and spleen showed lymphocyte death and loss of organ substructure in untreated but not VLP-treated mice (Fig. 2C-D). Although lymphocyte apoptosis was once thought to contribute to the pathogenesis of filovirus infection, recent studies indicate that inhibition of lymphocyte apoptosis does not ameliorate EBOV disease pathogenesis [37]; nonetheless, lymphocyte apoptosis still correlates with lethal infection [38]. Although TUNEL labeling was variable in each of the groups throughout infection, in general TUNEL labeling was increased in untreated mice when compared to VLP-treated mice (Fig. 2E).

### Viremia and systemic cytokine expression are reduced in VLP-treated mice

**Fig 3:**
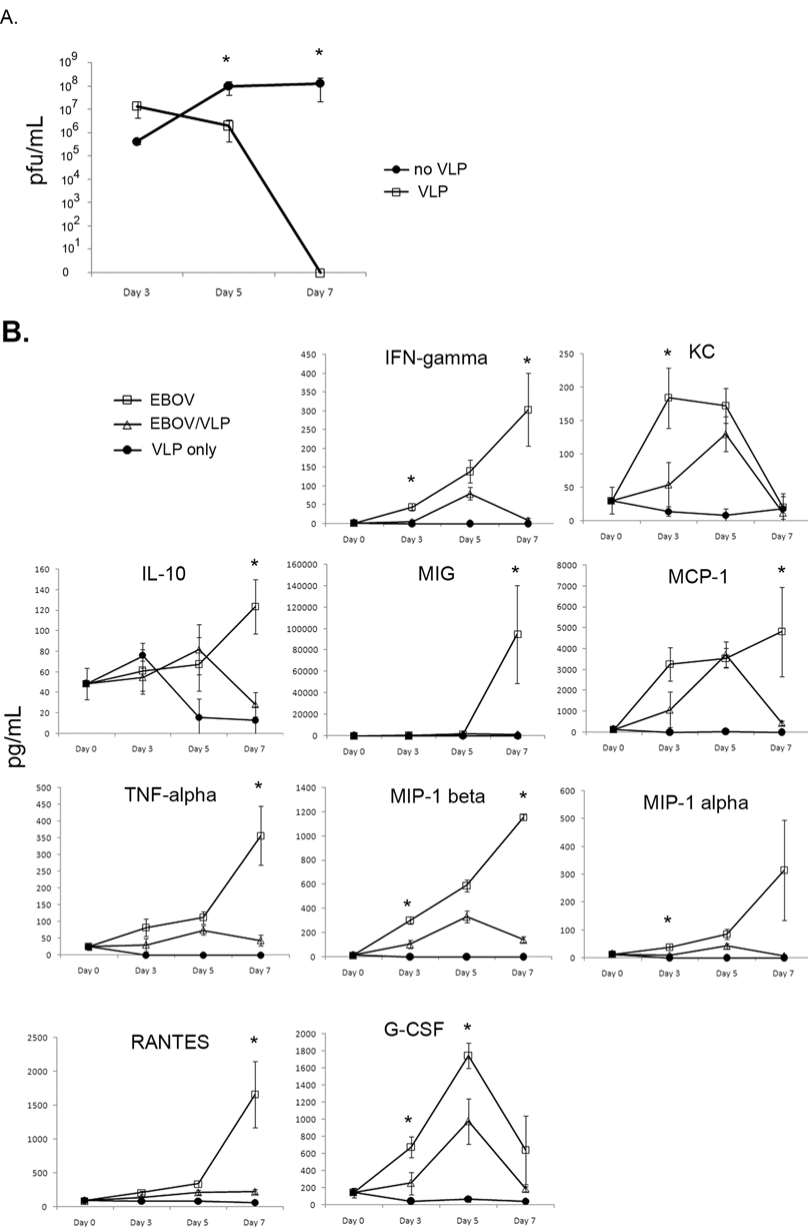
Viremia and serum cytokine levels are reduced in VLP-treated mice. **(A)** Serum from untreated or VLP-treated EBOV-infected mice was analyzed for viral plaque-forming activity. n = 7–8, two separate experiments. * p < 0.05. **(B)** Serum was collected from three groups of mice: uninfected, VLP-treated; EBOV-infected; and EBOV-infected, VLP-treated (24 hours after infection). Cytokine and chemokine levels were determined by using a cytometric bead array (CBA) flex set analyzing 21 cytokines and chemokines. n = 5–9 for EBOV-infected and EBOV-infected/VLP-treated groups (two separate experiments), n = 3 for VLP-treated, uninfected (one experiment). * p<0.05 between EBOV-infected and EBOV-infected/VLP-treated groups.

To attempt to discover the mechanisms of protection provided by post-exposure VLP treatment, serum was harvested at multiple time points and analyzed for viremia. As shown in Fig. 3A, viremia was similar at day 3 in both VLP-treated and untreated EBOV-infected mice (p>0.05), suggesting that VLP treatment does not inhibit early systemic viral replication. However, by day 5, there was a statistically significant decrease in viremia in the VLP-treated mice (p<0.01). Viremia was completely absent in Day 7 VLP-treated mice, while untreated mice had very high virus levels (p<0.01), correlating with Day 7 EBOV antigen staining in tissues (Fig. 2A-B).

To analyze systemic cytokine expression, serum was collected on Days 0, 3, 5, and 7 from uninfected mice treated with VLPs, EBOV-infected but untreated mice, or EBOV-infected mice treated with VLPs 24 hours post-infection. Statistically significant differences between EBOV-infected untreated mice and EBOV-infected VLP-treated mice were observed in 10 of 21 cytokines and chemokines tested (IFN-gamma, IL-10, TNF alpha, MCP-1, MIP-1 alpha, MIP-1 beta, RANTES, MIG, KC (orthologue to human IL-8), and G-CSF). In every case, these were elevated at some point in the untreated, EBOV-infected mice compared to the infected, VLP-treated mice (Fig. 3B). KC, G-CSF, MIP-1 alpha, MIP-1 beta, and IFN-gamma levels were elevated as early as day 3, whereas IL-10, MIG, MCP-1, TNF alpha, and RANTES levels were elevated on day 7. There was no statistical difference at any time point between untreated EBOV-infected mice and VLP-treated EBOV-infected mice for many other cytokines, including IL-2, IL-3, IL-4, IL-9, IL-12/23p40, IL-21, GM-CSF, or CD62L serum levels (S3 Fig.).

### B cells and perforin are required for VLP-mediated post-exposure protection

**Fig 4:**
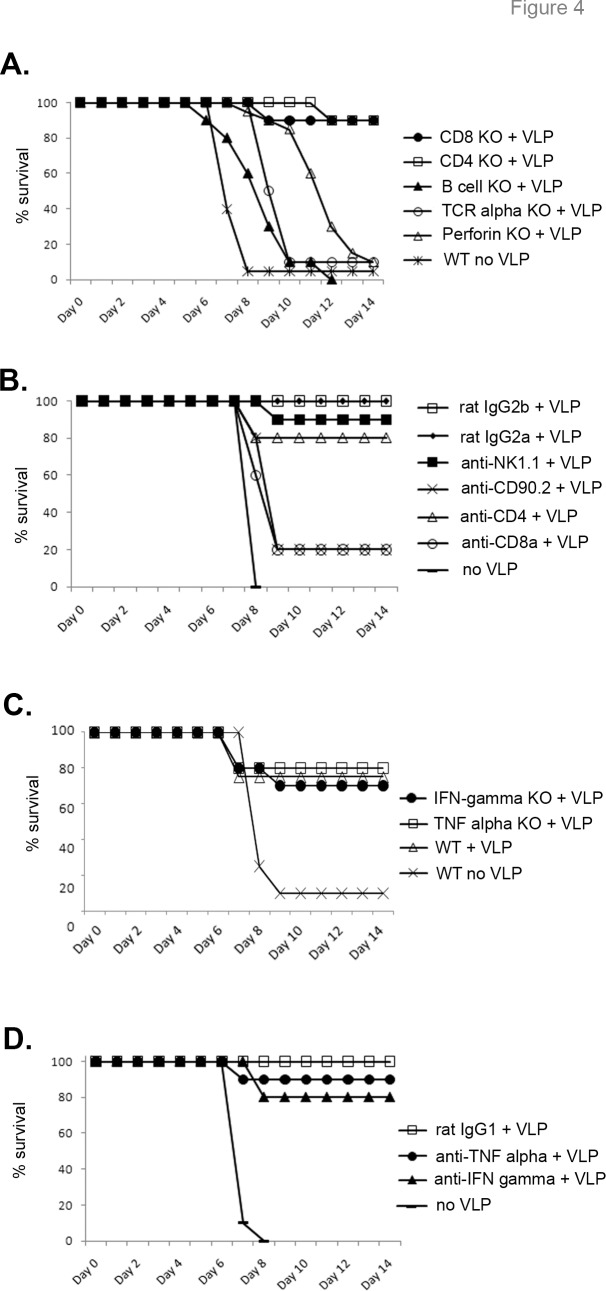
Immune components required for post-exposure VLP protection. **(A)** Various knockout mice were infected with EBOV and treated with VLPs 24 hours later. n = 10 (n = 20 for perforin and no VLP), 2 separate experiments. Nineteen of out 20 VLP treated control WT mice survived infection. (**B)** Wild-type mice were depleted of various cell types using depleting antibody treatment, infected with EBOV, and treated with VLP 24 hours after infection. Additionally, isotype controls for each antibody type used were tested. n = 5 (n = 10 for anti-NK1.1). **(C)** Mice lacking IFN-gamma or TNF-alpha were infected with EBOV and treated with VLP 24 hours later. n = 10 for IFN-gamma KO, n = 5 for TNF-alpha KO, n = 4 for WT, n = 20 for WT no VLP. **(D)** Wild-type mice were depleted of cytokines using depleting antibody treatment, infected with EBOV, and treated with VLP 24 hours after infection. Additionally, isotype controls for each antibody type used were tested. n = 5 (n = 10 for anti-TNF-alpha).

In order to further determine mechanisms of immune protection for the VLP-mediated post-exposure protection, various knockout (KO) mice were infected with EBOV and treated with VLPs 24 hours later. Mice lacking B cells were not protected by VLP treatment, whereas mice lacking either CD8+ or CD4+ T cells survived (Fig. 4A). However, mice lacking both CD8+ and CD4+ T cells (TCRa KO mice) were not protected by VLPs, suggesting that either CD8+ or CD4+ T cells are sufficient for protection, but neither subset is absolutely required. Interestingly, VLP-treated perforin knockout mice succumbed to EBOV infection, but with a delayed time-to-death relative to infected, untreated control mice (Fig. 4A). These data suggest that B cells and T cell, perforin-mediated killing is required for protection.

To corroborate the knockout mouse findings, antibody depletion studies were carried out in wild-type mice using published methods (see Materials and Methods). Confirming the knockout mouse data, depletion of CD4+ T cells did not abrogate VLP-mediated post-exposure protection, whereas depletion of both CD8+ and CD4+ T cells (using a CD90.2 antibody) eliminated protection (Fig. 4B). Additionally, depletion of NK cells using antibody showed that NK cells were not required for VLP-mediated protection. Depletion of CD8+ T cells (using an anti-CD8a antibody) did eliminate VLP-mediated post-exposure protection (Fig. 4B), seemingly contradicting what was observed in CD8a knockout mice (Fig. 4A). However, it has been reported that anti-CD8a antibody treatment can also deplete CD8a+ dendritic cells (DC) [39, 40]. Indeed, we found that the our protocol also depleted CD8a+ DC (>98% depletion of splenic CD8a+ DCs (S4 Fig.)), suggesting that depleting both CD8+ T cells and CD8a+ dendritic cells abrogates VLP postexposure protection.

To analyze the role of different cytokines in VLP-mediated post-exposure protection, mice lacking either IFN-gamma or TNF-alpha were infected and treated with VLPs. Interestingly, neither IFN-gamma nor TNF-alpha were required for protection (Fig. 4C). Antibody depletion of IFN-gamma or TNF-alpha confirmed the knockout mouse data that these cytokines are not required for VLP-mediated protection (Fig. 4D).

In order to determine whether appropriate memory responses were generated in the VLP-treated CD4+, CD8+, IFN-gamma, or TNF alpha knockout (or antibody depleted) mice that survived infection, these mice were re-challenged with EBOV >28 days later, without additional VLP treatment. All animals survived the second challenge without VLP treatment, and no animals displayed illness, suggesting that memory formation was sufficient for protection against subsequent challenge in these knockout mice (S1 Table). Additionally, these mice cleared the virus after re-challenge, as no viremia was detected >28 days post-re-challenge (S1 Table).

### Antibody levels are increased in VLP-treated mice

**Fig 5:**
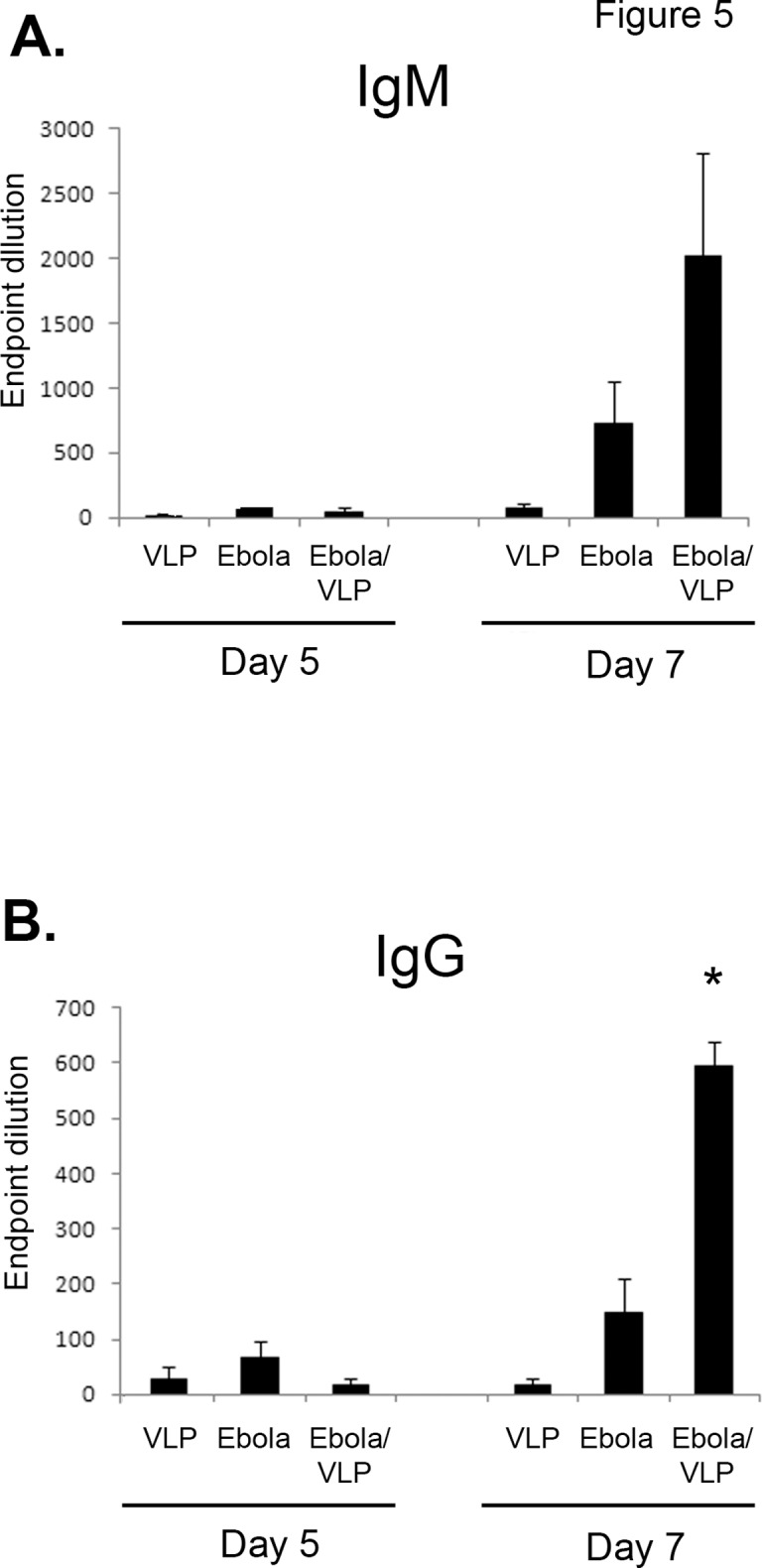
Antibody titers are increased after post-exposure VLP treatment. Serum from three groups of mice was collected and assayed for anti-EBOV antibodies: uninfected mice receiving VLPs only; EBOV-infected mice; and EBOV-infected mice treated with VLP 24 hours after infection. **(A)** IgM responses in mice. Although there was an increase in Day 7 EBOV-infected VLP-treated mice over EBOV-infected untreated mice, this difference was not statistically significant. **(B).** IgG responses. Note the increased IgG levels in EBOV-infected VLP-treated mice on Day 7. n = 7 (two separate experiments). * p<0.05 (EBOV-infected VLP-treated vs. EBOV-infected untreated).

Since B cells are required for VLP-mediated protection, serum antibody levels against EBOV antigen were measured. While it has been suggested that adaptive immune responses are ablated by EBOV virus-induced lymphocyte apoptosis, it has been recently been shown that CD8+ T cell responses are generated in lethal infection [41] and that inhibition of lymphocyte apoptosis does not ameliorate the disease course [37]. Additionally, a review of published literature revealed that 46% of lethal EBOV and Sudan ebolavirus human cases generated IgM responses to the virus, and 30% generated IgG responses prior to succumbing to disease [42–46]. As shown in Fig. 5, no significant differences in anti-EBOV IgM or IgG antibody titers were seen on day 5. However, EBOV-infected mice receiving VLPs had much higher day 7 EBOV-specific IgG levels compared to EBOV-infected, untreated mice or mice receiving only VLPs (Fig. 5B). IgM titers were also higher on day 7 in VLP-treated infected mice, but this difference was not statistically significant (Fig. 5A).

### Conventional dendritic cells and macrophages are required for VLP-mediated protection

**Fig 6:**
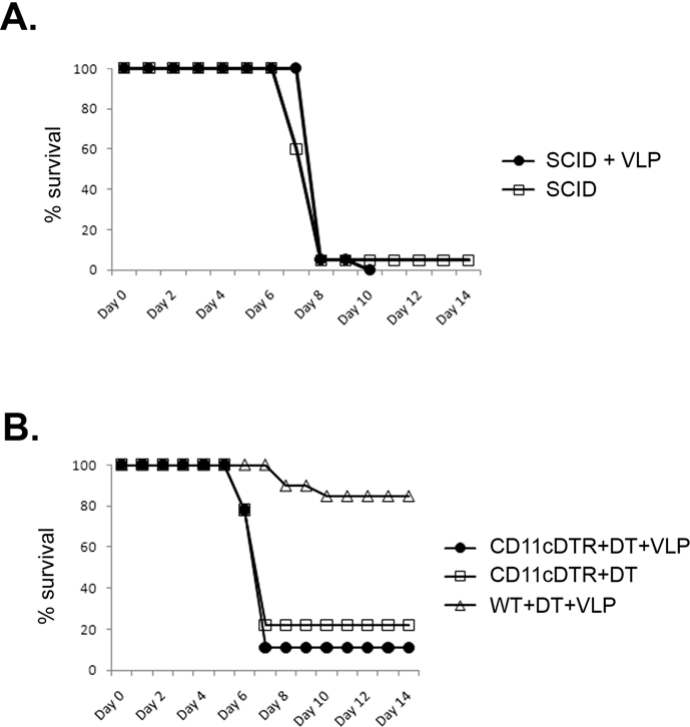
Conventional dendritic cells are required for VLP-mediated post-exposure protection. (**A)** SCID mice were infected with EBOV and treated with VLPs 24 hours after infection or left untreated. Little delay in time-to-death was observed with VLP treatment. n = 20, two separate experiments. **(B)** CD11c-DTR mice were injected with diphtheria toxin to deplete cDCs, then infected with EBOV and treated with VLP 24 hours after infection or left untreated. As a control, wild-type mice received diphtheria toxin injections and were treated with VLPs after infection. n = 9 for CD11c-DTR groups, n = 20 for WT (two separate experiments).

Since B cells and either CD4+ or CD8+ cells were required for protection, the role of innate cells was examined. As expected, SCID mice, lacking both B and T cells, were not protected by post-exposure VLP treatment (Fig. 6A). Additionally, there was only a miniscule increase in time to death in the VLP-treated group (7.58 ± 0.51 days in untreated mice vs. 8.10 ± 0.48 in VLP-treated mice), suggesting that any protective responses required the presence of an adaptive immune component, and that innate immune cells alone were not sufficient to mediate protection or delay time-to-death.

Next, the role of dendritic cells in VLP protection was assessed. Injection of diphtheria toxin (DT) into mice expressing the human DT receptor behind the CD11c promoter (CD11c-DTR mice) transiently eliminates most “conventional” DCs (cDC), but not plasmacytoid DCs [31]. Mice were depleted of cDC with DT injection, then infected with EBOV and either treated with VLP 24 hours after infection or left untreated. Depletion of cDCs abrogated VLP-mediated protection, whereas control wild-type mice receiving DT and VLP were protected (Fig. 6B). Additionally, depletion of cDCs in untreated, infected CD11c-DTR mice did not alter the lethality or time to death of EBOV (Fig. 6B), suggesting that cDCs, although putative sites of EBOV replication, are not required for EBOV-mediated pathogenesis.

**Fig 7:**
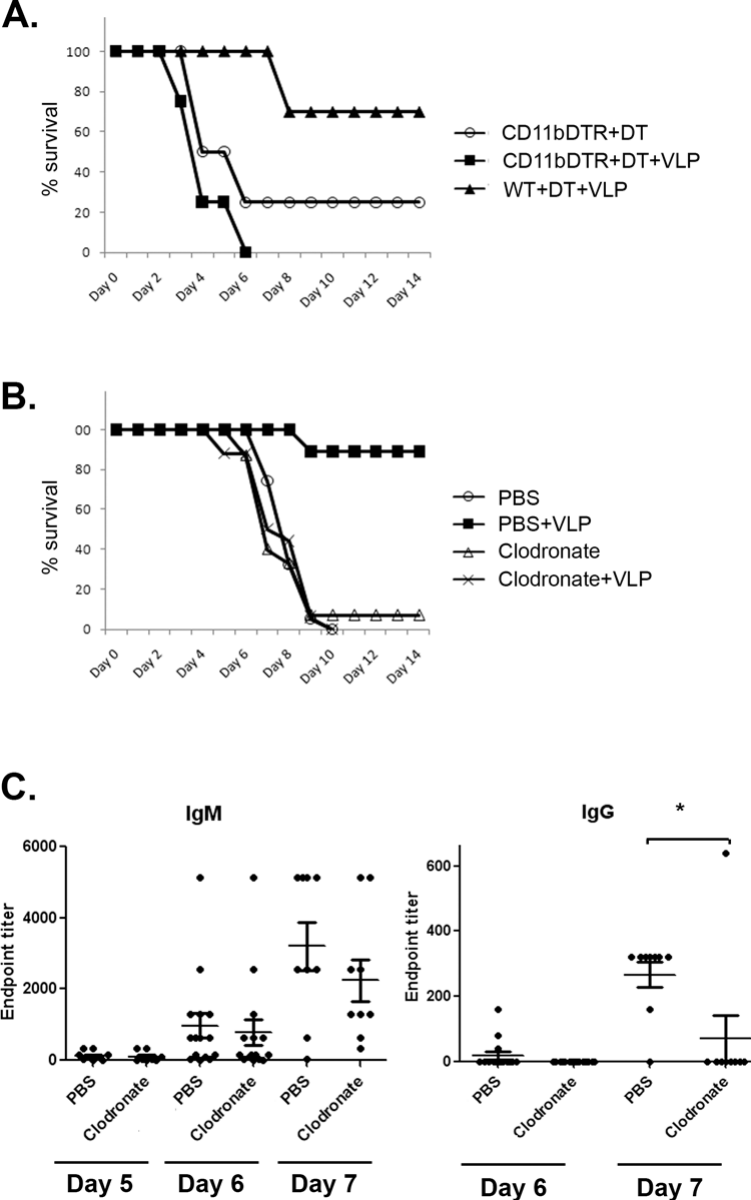
Macrophages are required for VLP-mediated post-exposure protection and IgG antibody production. **(A)** CD11b-DTR mice were depleted of macrophages by injection of diphtheria toxin, then infected with EBOV and treated with VLPs 24 hours post-infection, or left untreated. As a control, wild-type mice were treated with diphtheria toxin and treated with VLP after infection. n = 4 for CD11bDTR+DT and CD11bDTR+DT+VLP groups, n = 10 for WT+DT+VLP group. **(B)** Mice were depleted of macrophages using clodronate encapsulated liposomes, then infected with EBOV and treated with VLPs 24 hours after infection. As a control, mice received PBS encapsulated liposomes before infection and VLP treatment. Additional controls include PBS- or clodronate-treated mice without VLP treatment. n = 19 for PBS; n = 20 for PBS+VLP; n = 15 for Clodronate; n = 16 for Clodronate+VLP; two separate experiments. **(C)** Clodronate-treated mice (or control PBS-treated mice) were infected with EBOV and treated with VLPs. On days 5, 6, or 7, sera were analyzed for anti-EBOV IgG and IgM responses.

To assess the role of macrophages in VLP-mediated post-exposure protection against EBOV, two different methods were used. First, DT was injected into CD11b-DTR mice to transiently deplete CD11b-expressing cells (mostly macrophages) [32, 33]. Depletion of macrophages abrogated VLP-mediated protection (Fig. 7A). Another well-described method of depleting macrophages and other phagocytes is the use of clodronate-encapsulated liposomes (reviewed in [34]). Elimination of phagocytes in this manner also eliminated VLP-mediated protection, whereas mice receiving control PBS-encapsulated liposomes were protected (Fig. 7B). It is interesting to note that similar to cDCs, elimination of macrophages, which are thought to be early sites of filovirus replication [36, 47–53], does not reduce EBOV lethality or time to death in untreated, infected mice (Fig. 7A and B). Interestingly, ablation of phagocytes with clodronate abrogated the day 7 anti-EBOV IgG response found in EBOV-infected, VLP-treated mice (8/9 mice having no detectable IgG), while IgM production was unaffected (Fig. 7C).

## Discussion

### Mechanisms of immune protection in viral post-infection vaccination

Much of the literature describing immunological mechanisms utilized in post-exposure vaccination against viral infections centers on chronic infections; less work has been done on acute viral infections. However, a study using an acute, lethal mouse model of poxvirus infection indicates that post-exposure vaccine protection requires NK cells, CD4+ T cells, CD8+ T cells, and antibody; protection correlated with increased MCP-1, IL-6, MCP-3, IL-18, RANTES, and IFN-gamma production in serum [54]. These findings contrast almost completely with the filovirus work described here, where neither NK cells, CD4+ T cells, nor CD8+ T cells are required for protection, and decreases in pro-inflammatory cytokine levels in VLP-treated versus untreated mice were found. Interestingly, however, both viruses required B cells for protection. It is clear that mechanisms of post-exposure vaccination protection will vary depending on the nature of the virus. Additionally the VLP platform is non-replicating, which varies considerably from a replicating vaccine such as those used for poxviruses. Given the dearth of published information on post-exposure mechanisms of immune protection in acute viral infections, the data reported here and in the companion paper significantly add to this field.

### Role of immune components in post-exposure EBOV protection

CD4+ or CD8+ T cell knockout mice were protected by the VLPs, which originally suggested a lack of importance of the T cell compartment in VLP-mediated post-exposure protection. However, additional studies showed that mice lacking both CD4+ and CD8+ T cell subsets succumbed to infection (Fig. 4A). Also, mice lacking perforin were not rescued with VLP treatment and succumbed with a delayed time-to-death, whereas depletion of NK cells did not abrogate protection (Fig. 4A). Therefore, the perforin requirement was not due to NK function. One interpretation of this data is that at least one T cell subset is needed to act as perforin-dependent cytotoxic cells to control infection. CD4+ T cells can act as cytotoxic T cells in certain viral infections [55], and we hypothesize that CD4+ cytotoxic T cells may be generated in post-exposure VLP-treated mice, at least in the absence of CD8+ T cells. Surprisingly, mice deficient in IFN-gamma or TNF-alpha are protected by post-exposure VLP treatment (Fig. 4B).

B cells were also required for protection (Fig. 4A), and increased antibody titers correlated with protection in VLP-treated versus untreated mice (Fig. 5 and Fig. 7C). These data correlate with the need for GP in the VLP preparation for protection (Fig. 1D). Interestingly, VLPs consisting of GP, NP, and VP40 were protective, whereas VLPs lacking GP (either NP/VP40 or VP40 only VLPs) did not provide protection when given post-exposure (Fig. 1D). Presumably this is due to the lack of an appropriate antibody target (GP). Indeed, post-exposure transfer of antibodies against GP have been shown to protect nonhuman primates from EBOV infection [16–20], and antibody responses are correlated with or required for protection in multiple, but not all, pre-exposure filovirus vaccine studies [24, 56–58]. Furthermore, most effective EBOV vaccines in animal models utilize GP as the primary immunogen, and a GP-only pre-exposure subunit vaccine is protective in mouse studies [59].

Interestingly, VLP-treated mice lacking CD4+ T cells were protected from EBOV infection, whereas B cell knockout mice succumbed (Fig. 4A). This suggests that T-independent B cell responses may be sufficient for mediating protection. Although it is likely that pre-exposure vaccination with VLPs do induce T-dependent responses [24], additional studies are required to determine the mechanisms of B cell subset involvement (including marginal zone B cells) in post-exposure VLP vaccination.

It was surprising to note that day 7 antibody titers were higher in EBOV-infected, untreated mice than in VLP-treated, uninfected mice (Fig. 5). It is possible that this is due to increased antigen availability in infected animals, leading to higher antibody titers. Another possibility is that defective interfering (DI) particles generated during the replication process [60] serve as stimulators of innate (i.e. type I IFN) and subsequently adaptive immunity. This would also explain why VLPs given during EBOV infection generate higher levels of antibody than VLPs alone or EBOV infection alone.

Although depletion of cDC or macrophages abrogated VLP-mediated post-infection protection, VLP treatment did not extend time-to-death in SCID mice (Fig. 6A). This suggests that although cDCs (Fig. 6B) and macrophages (Fig. 7A and 7B) are required for post-exposure VLP protection, their functionality against EBOV requires interactions with B and T cells. This is further shown by the finding that antibody responses were diminished in VLP-treated macrophage-depleted mice (Fig. 7C).

### DCs and macrophages in EBOV pathogenesis

It is important to note that mice depleted of cDCs (Fig. 6B) or macrophages (Fig. 7A and 7B) still succumb to EBOV infection, regardless of the presence or absence of VLP treatment, even though there is speculation that DCs and monocytes/macrophages are early primary targets for filovirus replication and are important for viral spread [61]. The data presented here show that macrophages and cDCs are required for VLP-mediated post-exposure protection, but are not required for EBOV replication and subsequent pathogenesis. Other cell types must also be able to serve as sites of early EBOV infection and replication.

### Pre-exposure versus post-exposure mechanisms of immune protection and vaccine component requirements

Despite the well-described use of VLPs as a pre-exposure vaccine for filovirus infections (reviewed in [62]), no published studies have focused on post-exposure therapeutic use of VLPs for EBOV infection. In the post-exposure model reported here, B cells are required for protection (Fig. 4A), and VLP treatment augments antibody titers by day 7 in infected mice (Fig. 5B). Pre-exposure vaccination with VLP requires B cells for protection against EBOV in mice [9, 24], so B cells are required for both pre-and post-exposure VLP efficacy. VP40 only VLP, when given 3 days prior to infection, is sufficient to protect mice from low-dose EBOV challenge [35], but is not protective post-exposure (Fig. 1D). Additionally, VLPs administered 3 days before infection require NK cells for protection [35], but NK cells are dispensable in post-exposure treatment (Fig. 4D). Previous reports indicate that CD8+ T cells and IFN-gamma are required for pre-exposure VLP-mediated protection (i.e. challenge 6 weeks after second vaccination), whereas perforin is not required [24]; the opposite is found in post-exposure VLP treatment reported here. Interestingly, adenovirus-based EBOV vaccines require B cells, but not IFN-gamma for pre-exposure protection, while the role of CD8+ T cells is controversial [63–64]. VSV-based vaccines do not require CD8+ T cells for pre-exposure protection [65]. These results suggest that the immune responses required for protection may differ based on the vaccine platform and time of viral challenge relative to vaccination, a finding that should be considered when designing pre- versus post-exposure therapeutics. However, it is problematic to directly compare pre-exposure requirements and post-exposure requirements, even within the same viral platform. Pre-exposure vaccination requirements are tested by the formation of memory immune responses, whereas post-exposure vaccination utilizes primary immune responses. Additionally, different vaccine dosage and adjuvant formulation may induce varied immune responses.

Overall, however, B cells have been shown to be required for protection in multiple pre-exposure filovirus vaccine studies, as well as the post-exposure model described here. Given the recent success in antibody-based therapies against filoviruses, it is likely that B cells are required for vaccine efficacy regardless of the platform used.

The route of VLP administration appeared to affect post-exposure efficacy, as intraperitoneal vaccination was more effective at lower doses than intramuscular vaccination. Both routes are effective in pre-exposure protection [9], suggesting that route-of-delivery efficacy is integral to timing of administration relative to infection.

Importantly, we show that the post-exposure treatment was not due to non-specific induction of innate responses, since marburgvirus VLPs did not protect mice from EBOV challenge (Fig. 1A), and increased EBOV specific antibodies were found in VLP treated vs untreated mice (Fig. 5B).

### A model of immune mechanisms of VLP-mediated post-exposure protection against EBOV

In a companion paper [69], we demonstrate that VLP post-exposure protection is also dependent on type I IFN signaling. VLP treatment after EBOV infection induces increased early type I IFN responses, including interferon stimulating genes (ISGs) with anti-viral and anti-inflammatory functions. Mice lacking type I IFN signaling have increased pro-inflammatory cytokine production after EBOV infection, and succumb even with VLP post-exposure treatment, while VLP-treated wild-type mice have diminished proinflammatory cytokine production.

Together our data suggest a model in which post-exposure VLP treatment induces early type I IFN responses that dampen excessive inflammatory cytokine production and limit organ damage. Indeed, high levels of proinflammatory cytokines are linked with lethal disease in non-human primate [63, 66] and human studies [67–68]. Subsequently, our model indicates that increased antibody responses are generated (dependent on macrophages) which limit EBOV replication until cytotoxic T cells resolve infection (Figure 8 in [69]). VLP post-exposure treatment requires that innate and adaptive immune responses work in concert, as VLPs do not improve survival or time-to-death in SCID mice. Together, these data provide an in-depth analysis of mechanisms of immunity in VLP-mediated post-exposure protection against EBOV infection.

## Supporting Information

S1 Fig Histopathology on Day 3 post-infection. (TIF)

S2 Fig Histopathology on Day 5 post-infection. (TIF)

S3 Fig Cytokine levels in sera. (TIF)

S4 Fig Depletion of CD8a+ DCs. (TIF)

S1 Table Surviving VLP-treated mice are protected from subsequent rechallenge. (DOCX)
