# Supplementary figures and images for: Mechanisms of Immunity in Post-Exposure Vaccination against Ebola Virus Infection
Source: PLoS One. 2015 Mar 18;10(3):e0118434. doi: 10.1371/journal.pone.0118434 (PMC4364937; doi:10.1371/journal.pone.0118434)

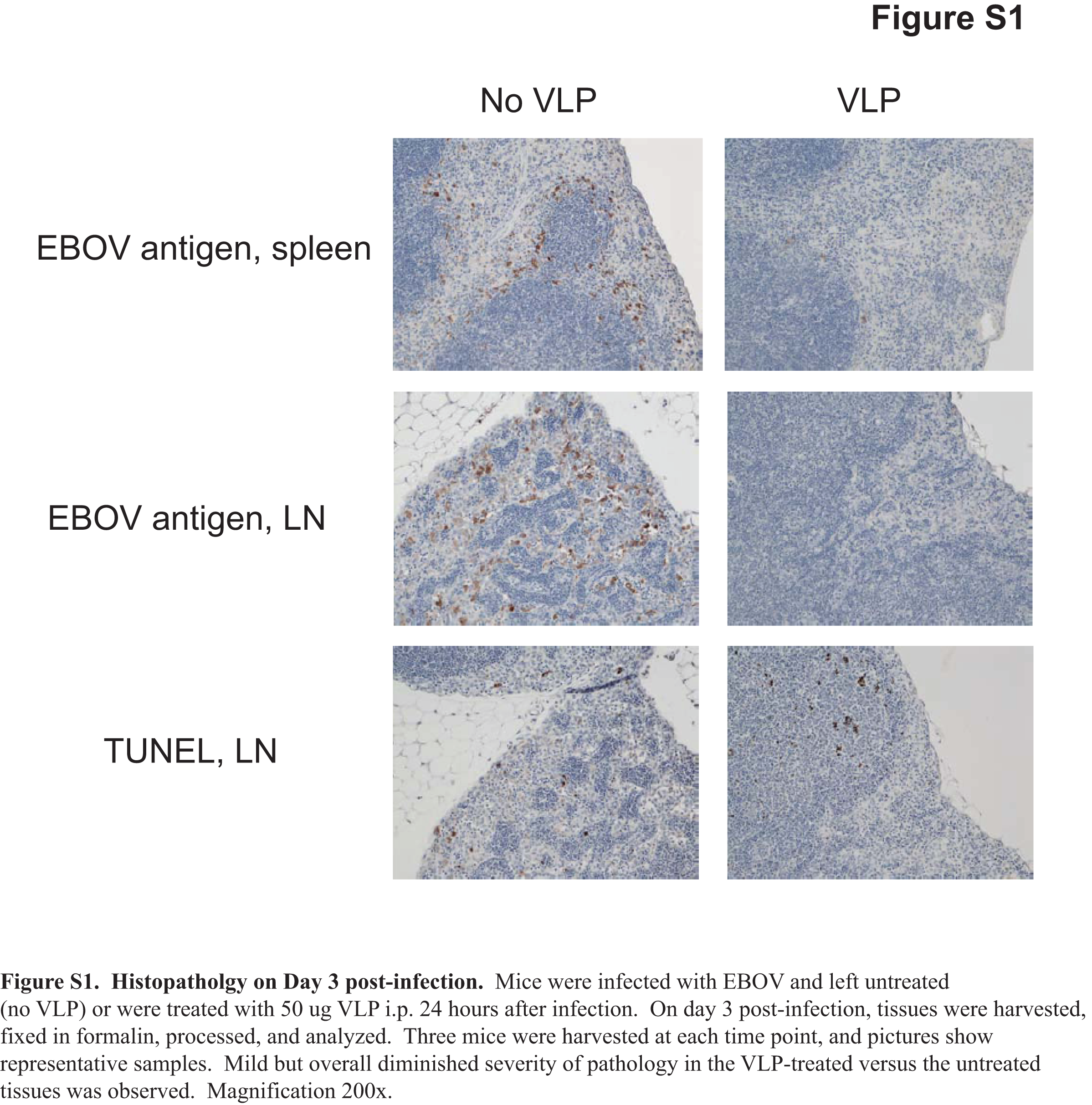

Supplement: S1 Fig — (TIF) [file pone.0118434.s001.tif]

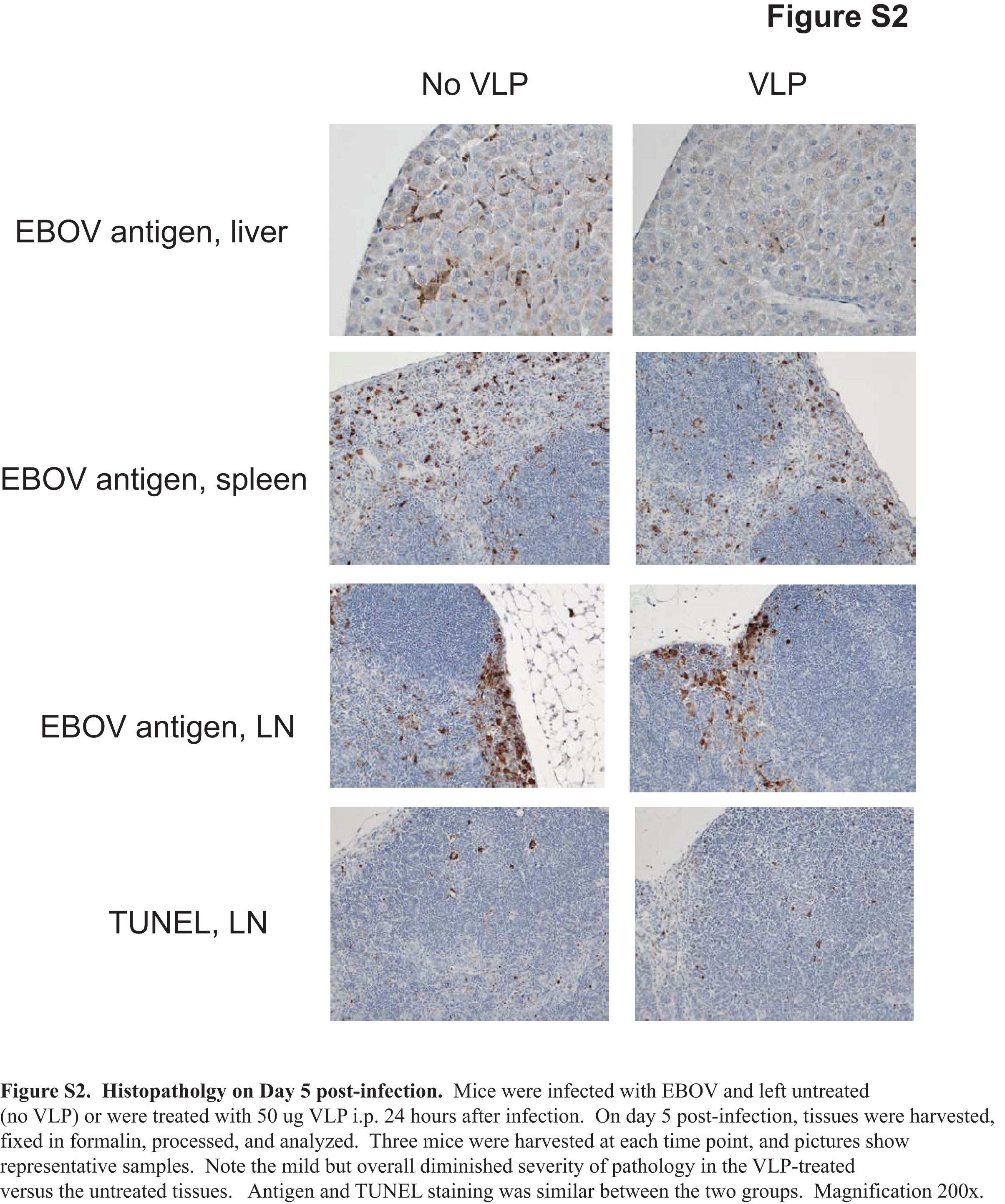

Supplement: S2 Fig — (TIF) [file pone.0118434.s002.tif]

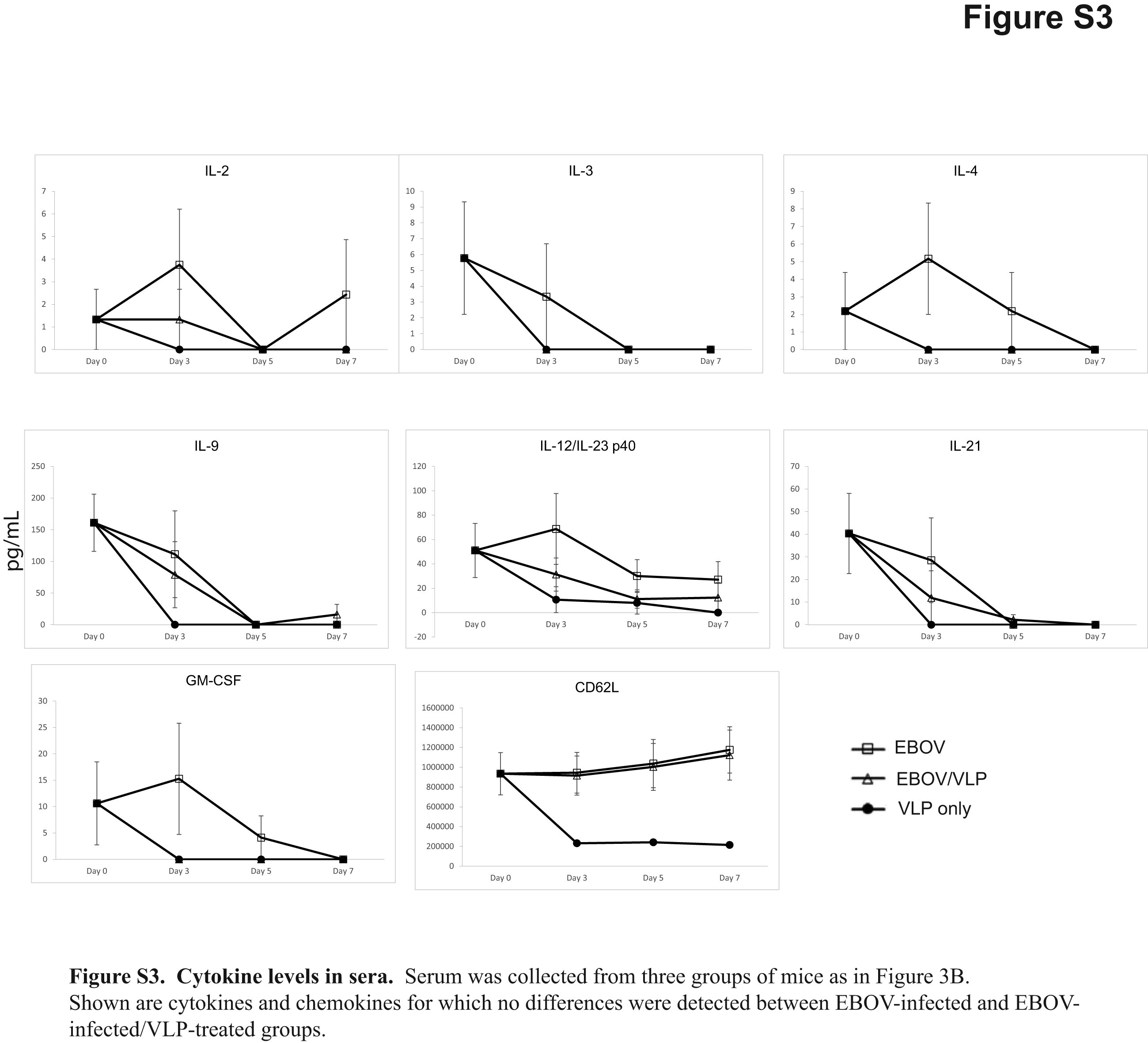

Supplement: S3 Fig — (TIF) [file pone.0118434.s003.tif]

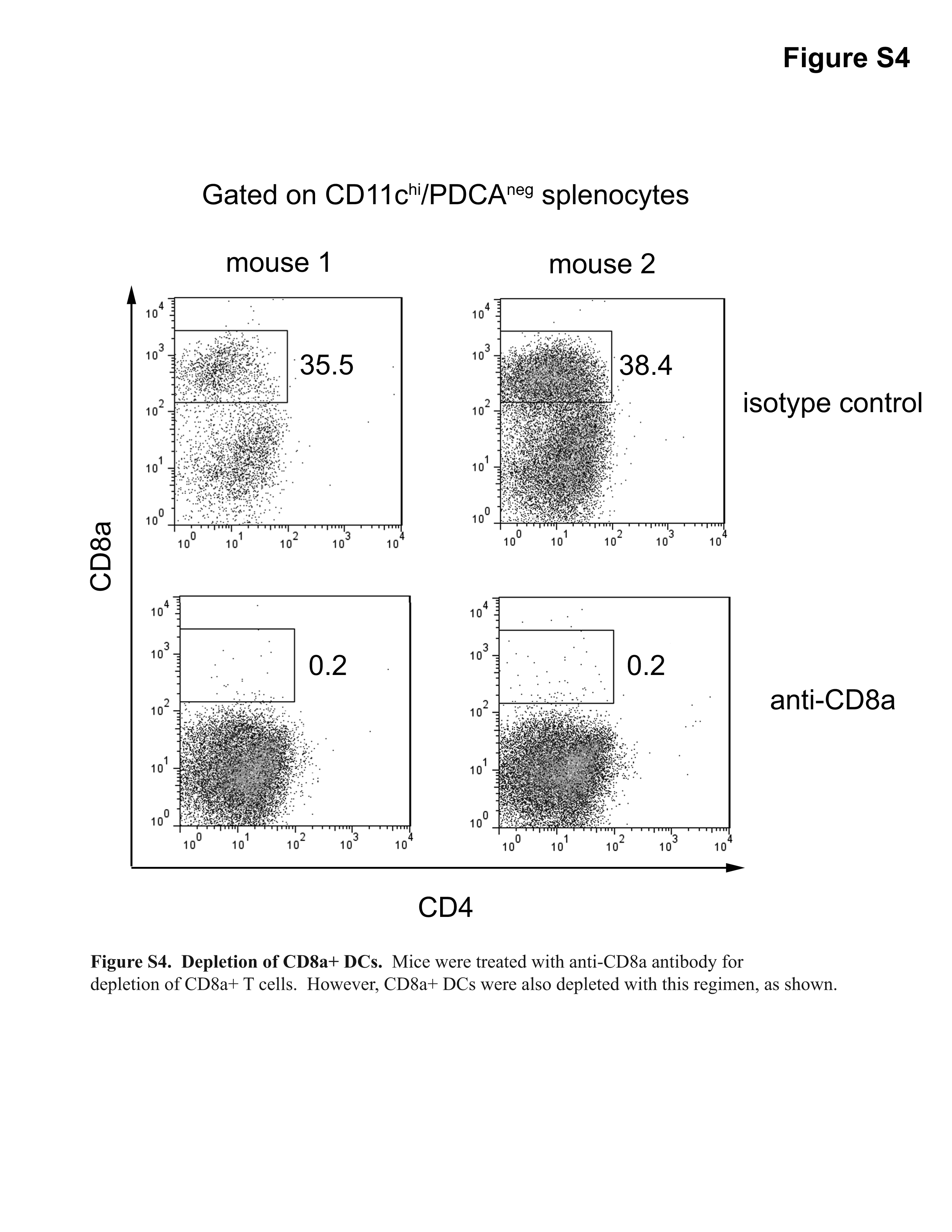

Supplement: S4 Fig — (TIF) [file pone.0118434.s004.tif]
