# Supplementary material for: Mechanisms of Immunity in Post-Exposure Vaccination against Ebola Virus Infection
Source: PLoS One. 2015 Mar 18;10(3):e0118434. doi: 10.1371/journal.pone.0118434 (PMC4364937; doi:10.1371/journal.pone.0118434)
Supplement: S1 Table — (DOCX) [file pone.0118434.s005.docx]

| **Mice** | **Survival after re-challenge** | **Viremia >28 days post re-challenge** |
| --- | --- | --- |
| CD4 KO | 9/9 | None detected |
| CD8 KO | 9/9 | None detected |
| IFN-gamma KO | 7/7 | None detected |
| TNF-alpha KO | 4/4 | None detected |
| CD4 depleted | 4/4 | None detected |
| IFN-gamma depleted | 4/4 | None detected |
| TNF-alpha depleted | 9/9 | None detected |
| NK1.1 depleted | 9/9 | None detected |

**Table S1. Surviving VLP-treated mice are protected from subsequent rechallenge.** Mice from Figure 4 that survived infection were rechallenged >28 days after initial infection. All challenged mice survived, and viremia was not detected in any animal >28 days post-rechallenge. Control naïve WT mice succumbed to infection (18/20).
